# Radiation Therapy in Addition to Gross Total Resection of Retroperitoneal Sarcoma Results in Prolonged Survival: Results from a Single Institutional Study

**DOI:** 10.1155/2008/824036

**Published:** 2009-01-29

**Authors:** Timothy M. Zagar, Robert R. Shenk, Julian A. Kim, Deb Harpp, Charles A. Kunos, Fadi W. Abdul-Karim, William C. Chen, Yuji Seo, Timothy J. Kinsella

**Affiliations:** ^1^Department of Radiation Oncology, Case Medical Center, University Hospitals, 11100 Euclid Avenue, Cleveland, OH 44106, USA; ^2^Department of Surgery, Case Medical Center, University Hospitals, 11100 Euclid Avenue, Cleveland, OH 44106, USA; ^3^Department of Pathology, Case Medical Center, University Hospitals, 11100 Euclid Avenue, Cleveland, OH 44106, USA; ^4^Department of Radiation Oncology, School of Medicine, Case Western Reserve University, Biomedical Research Building, Room 326, 2109 Adelbert Road, Cleveland, OH 44106, USA

## Abstract

*Purpose*. Typical treatment of retroperitoneal sarcomas (RPSs) is surgery with or without radiation therapy for localized disease. With surgery alone, local failure rates are as high as 90%; this led to radiation therapy playing an important role in the treatment of RPSs. *Methods*. Thirty-one patients with retroperitoneal sarcoma treated with gross total resection and radiation therapy make up this retrospective analysis. Nineteen were treated preoperatively and 12 postoperatively (median dose, 59.4 Gy)—sixteen also received intraoperative radiation therapy (IORT) (median dose, 11 Gy). Patients were followed with stringent regimens, including frequent CT scans of the chest, abdomen, and pelvis. *Results*. With a median follow-up of 19 months (range 1–66 months), the 2-year overall survival (OS) rate is 70% (median, 52 months). The 2-year locoregional control (LRC) rate is 77% (median, 61.6 months). The 2-year distant disease free survival (DDFS) rate is 70% (median not reached). There were no differences in radiation-related acute and late toxicities among patients treated pre- versus postoperatively, whether with or without IORT. *Conclusions*. Compared to surgery alone, neoadjuvant or adjuvant radiation therapy offers patients with RPS an excellent chance for long-term LRC, DDS, and OS. The integration of modern treatment planning for external beam radiation therapy and IORT allows for higher doses to be delivered with acceptable toxicities.

## 1. Introduction

Retroperitoneal sarcomas (RPSs) make up a minority of soft tissue sarcomas (STSs) diagnosed in the United States. In 2008, it is estimated that 10 390 people will be diagnosed with an STS; of these, approximately 20%, or 2080, will occur in the retroperitoneum [1]. RPSs pose difficult diagnostic and therapeutic problems for physicians treating them. As a result of their location, sarcomas that arise in this area can grow to extremely large sizes (typically >10 cm) before causing symptoms. Their large masses may encompass critical anatomic structures, significantly decreasing the rate of complete surgical resectability [2–4]. Only approximately half of RPSs are surgically resectable. Without adjuvant therapy, the risk of local recurrence ranges from 68% to more than 90% at 10 years [5–7]. The prospect of adjuvant radiation therapy to “sterilize” positive surgical margins and/or minimal gross residual disease has been vital in the treatment of RPSs. Additionally, preoperative radiation therapy is often used in an attempt to improve resectability.

There has been no published randomized trial for preoperative versus postoperative radiation therapy in patients with RPS, as there has been for soft tissue sarcomas of the extremity [8], largely because of the paucity of cases. As such, there is no consensus as to the sequencing, or even the need for radiation therapy for that matter. Radiation therapy has been given in the preoperative setting [9–12], in the hopes of converting a technically unresectable RPS to being resectable. It has also been given in the preoperative [13] and postoperative setting with concurrent chemotherapy [14]. It can also be given in the postoperative setting to help control any residual disease, be it gross or microscopic [10, 11, 15–19]. Finally, radiation therapy can be given in the intraoperative setting either with electrons from a linear accelerator or with brachytherapy, both of which have been shown to increase local control [9, 10, 12, 16, 18–20].

At our institution, preoperative radiation therapy with or without intraoperative radiation therapy (IORT) has been utilized for large, potentially difficult-to-resect RPSs. Radiation therapy is recommended in the postoperative adjuvant setting in patients who had surgery prior to initial referral, or for smaller RPSs that our surgeons feel they can completely resect without having to sacrifice too much normal anatomy for the sake of achieving a gross total resection (GTR). We report one of the largest single institution cohorts of patients with RPS treated with modern radiation therapy techniques, including a subset direct comparison between those patients treated pre- versus postoperatively, to elucidate any differences in both outcome and toxicities.

## 2. Materials and Methods

### 2.1. Patients

Between August 2000 and April 2008, 29 consecutive adult patients with a histologic diagnosis of retroperitoneal sarcoma and 2 patients with recurrent/persistent intraabdominal aggressive fibromatosis (desmoid tumor) were treated with at least one modality of radiation therapy at Case Medical Center in University Hospitals. All patients were presented to the biweekly multidisciplinary sarcoma tumor board prior to treatment at our institution. Data were collected retrospectively from paper charts and electronic medical records after approval by our Cancer Center Review Board and our Institutional Review Board.

### 2.2. Treatment Regimens

#### 2.2.1. Pre- and Postoperative External Beam Radiotherapy

Radiation therapy planning utilizing standard computed tomography (CT)-guided 3-dimensional conformal techniques was routinely performed, typically with 4 to 7 fields used. As the techniques of intensity modulated (IMRT) and image-guided radiation therapy (IGRT) utilizing TomoTherapy (Madison, Wis, USA) became available in 2004, they were utilized as per the physician's discretion. All radiation treatment planning was performed by a senior radiation oncologist (TJK).

#### 2.2.2. Intraoperative Radiation Therapy (IORT)

When clinically indicated, IORT was administered via a Mobetron linear accelerator with electron energies of 6 to 12 MeV, prescribed to the 90% isodose line (Intraop Medical, Inc., Sunnyvale, Calif, USA). After surgical resection of the RPS, a flat or beveled cylindrical cone (5–10 cm) anchored by a Bookwalter surgical clamp (Codman and Shurtleff, Inc., Raynham, Mass, USA) was placed by both the surgeon and radiation oncologist over areas at high risk of residual microscopic to minimal gross disease. No patients were treated with IORT for unresectable disease. Dose-limiting normal tissues that were in the desired IORT bed, if mobile, were physically moved out of the field—if they were immobile, typically 3 half value layer lead wafers were used for shielding. The technique of IORT and surgical approaches to minimize acute and late IORT-related normal tissue toxicities are previously detailed [21, 22].

#### 2.2.3. Surgery

A gross total resection (GTR) was achieved when the surgeon was able to resect all areas of visible disease in the operating room—a subtotal resection (STR) was performed when there was any amount of gross residual disease left behind. An *en bloc* resection of the tumor mass and expendible involved normal tissues was attempted in all patients. Microscopic margin status was not routinely reported, but was assumed to be positive along soft tissue, bone, and vascular margins in the retroperitoneum.

#### 2.2.4. Follow-Up

Patients were followed up by their treating radiation oncologist after completing radiation every 3 months for the first year, every 4 months for the 2nd year, every 6 months for the 3rd and 4th years, then annually, with a contrast enhanced CT of the chest, abdomen, and pelvis, and full labs with each visit. Other ancillary studies were performed as clinically indicated.

#### 2.2.5. Normal Tissue Toxicity Analyses

Acute (1–90 days after radiation therapy commencement) and chronic (>90 days) toxicities were graded according to the Radiation Therapy Oncology Group (RTOG) acute and late radiation morbidity scoring criteria [23].

#### 2.2.6. Definition of Treatment Outcomes and Statistical Considerations

The primary endpoints of this study were locoregional control (LRC), distant disease-free survival (DDFS), and overall survival (OS). A locoregional failure was defined as any tumor recurrence within the abdominal/retroperitoneal cavity, including within radiation portals. Distant metastases (typically to liver and/or lung) were determined by CT examinations with or without subsequent histopathologic confirmation. OS, DDFS, and LRC were calculated from the first day of radiation therapy administration. The patients were censored at either their last follow-up, if they remained alive and/or disease free, or the date of their local or distant failure or death. The Kaplan-Meier method was used to compute LRC, DDFS, and OS [24]. Cox models were used for multivariate analysis of prognostic factors [25]. Chi square and Fisher's exact tests were utilized for categorical variables and median tests were utilized to analyze the continuous variables to compare patient groups treated pre- versus postoperatively [26]. All measures tests were analyzed with statistical software (SPSS 12.0, SPSS, Inc, Chicago, Ill, USA), with a 2-sided alpha level of 0.05 regarded as statistically significant.

## 3. Results

### 3.1. Patient and Treatment Characteristics

Twenty-nine consecutive patients with biopsy proven retroperitoneal sarcoma and 2 patients with intraabdominal fibromatosis (desmoid tumor) were treated and closely followed over this 8+ year period, for a median of 19 months (range 1–66 months). The 2 patients with large intraabdominal desmoid tumors were included in this analysis due to the highly aggressive nature of their lesions—in fact, one patient had positive lymph nodes at the time of surgical resection. Females made up the majority (65%) of patients, and Caucasians accounted for the overwhelming majority (90%). Most frequently, patients presented with either pain or some type of vague abdominal discomfort, or unintentional weight loss (Table 1). Overall, 26 (84%) patients were able to have a gross total resection of their RPSs; 15 (79%) of the patients were treated preoperatively and 11 (92%) of the patients were treated postoperatively. Patients treated postoperatively generally had smaller tumors (≤10 cm) at presentation. When separated into sequencing of treatment, 19 (61%) patients were treated preoperatively (median dose 59.4 Gy, range 36.8–63.4 Gy), with the goal of improving the likelihood of achieving gross total resection; one patient was palliated for her SVC syndrome, which was her presenting symptom. Twelve (39%) patients were treated in the postoperative setting (median 59.4 Gy, range 54–68.4 Gy) (Table 2). Both groups were well matched with respect to patient characteristics, pathology, grade, clinical stage, histology, and extent of surgical resection. However, the only statistically significant difference between the groups was the use of IORT and IMRT. Thirteen (68%) patients were treated with preoperative EBRT received IORT, compared to only 3 (25%) patients who received IORT followed by postoperative EBRT, based on the recommendations of the multidisciplinary sarcoma tumor board (*P* = .03, Table 2). Eight (67%) patients treated with postoperative EBRT were treated with IMRT/IGRT versus only 2 (11%) patients in the preoperative group, based on the availability of TomoTherapy (*P* = .003).

#### 3.1.1. Locoregional Control

**Figure 1:**
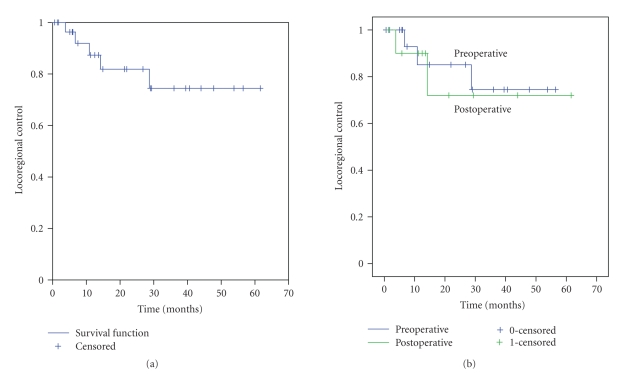
(a) Locoregional control: all 31 patients. (b) Locoregional control: pre- versus postoperative EBRT (*P* = .79). EBRT = external beam radiation therapy.

Overall, the 31 patients have a median locoregional control rate (LRC) of 61.6 months, with a 2-year LRC rate of 77% (SE +/− 18%) (Figure 1(a)). Two patients in the preoperative group and 1 patient in the postoperative group developed a locoregional failure as their first site of failure. There was no statistically significant difference in LRC rates in patients treated pre- versus postoperatively (*P* = .79) (Figure 1(b)). The use of IORT did not have an impact on LRC (*P* = .550) (Table 3).

#### 3.1.2. Distant Metastases

**Figure 2:**
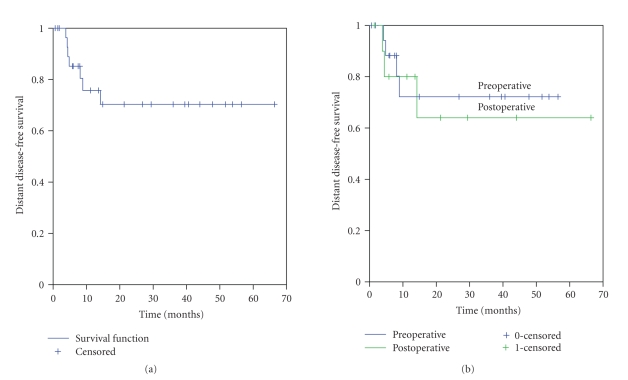
(a) Distant disease free survival: all 31 patients. (b) Distant disease free survival: pre- versus postoperative EBRT (*P* = .73). EBRT = external beam radiation therapy.

The median distant disease-free survival (DDFS) has not yet been achieved, with a 2-year DDFS of 70% (SE +/− 18%) (Figure 2(a)). All 7 patients who developed metastatic disease did so within the first 14 months of beginning radiation therapy (median 4.8 months, range 4.2–14.2 months). Treatment for metastases was physician dependent—all were offered chemotherapy. One patient underwent multiple video-assisted thorascopic (VATS) resections of his pulmonary metastases, while one other received palliative radiation therapy to her lung metastases. Four patients in the preoperative group and 2 patients in the postoperative group developed distant metastases as their first site of failure—two patients in the postoperative group developed synchronous distant and locoregional failures. There was no statistically significant difference in DDFS rates in patients treated pre- versus postoperatively (*P* = .73), whether with or without IORT (*P* = .42); see Figure 2(b) and Table 3.

#### 3.2. Overall Survival

**Figure 3:**
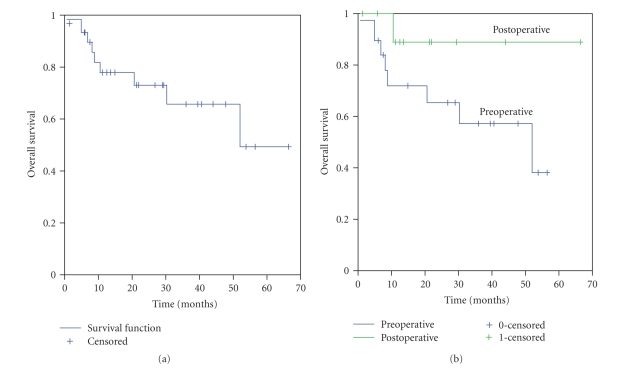
(a) Overall survival: all 31 patients. (b) Overall survival: pre- versus postoperative EBRT (*P* = .14). EBRT = external beam radiation therapy.

The median overall survival for this patient cohort was 52 months, with a 2-year OS rate of 70% (SE +/− 19%) (Figure 3(a)). Nine patients died during follow-up, with 8 of these directly attributable to retroperitoneal sarcoma. The other death occurred in a patient who suffered from a complicated postoperative course, including intractable clostridium difficile colitis. At last follow-up (5.8 months), she was free of both local and recurrent disease, but she was ultimately lost to follow-up. There was no statistically significant difference in DDFS rates in patients treated pre- versus postoperatively (*P* = .14), whether with or without IORT (*P* = .77); see Figure 3(b) and Table 3.

### 3.3. Prognostic Factors for LRC, DDFS, and OS

There were two trends that approached statistical significance; those patients treated postoperatively had a trend to have lesions ≤10 cm (*P* = .07), and were more likely to present with recurrent disease (*P* = .08). In our cohort of patients, after controlling for whether patients were treated in the preoperative or postoperative setting, none of the commonly cited characteristics had a statistically significant impact on LRC, DDFS, or OS, on multivariate analysis (Table 3).

#### 3.3.1. Toxicity

Most patients (90%) completed the planned course of radiation therapy without significant (≥grade 3) acute effects. Acute and late toxicities were similar between the pre- and postoperative groups (Table 4). There were 3 grade 3-4 acute radiation-related toxicities (all 3 in the preoperative group), and 17 grade 3-4 late toxicities (11 in the preoperative group, 6 in the postoperative group)—no grade 5 treatment-related toxicities occurred. The most common acute side effect seen, with almost 90% of patients overall experiencing, was grade 1-2 gastrointestinal complaints, most commonly being nausea and/or diarrhea. These grade 1-2 toxicities responded to conservative medical treatment. Late toxicities occurred less frequently and there were no statistically significant differences in late grade 3-4 toxicities between those patients treated pre- versus postoperatively (Table 4). Once again, late GI toxicities were the most common type of a late side effect—when they occurred, they were more often grade 3 to 4, including requiring emergency surgery for small bowel obstruction related to adhesions (one patient each in the preoperative and postoperative groups). There was one grade 1 (asymptomatic) jejunal stricture found at surgery for another reason, in a patient treated postoperatively, that was presumed to be radiation-related. One patient who was treated with postoperative radiation therapy for his second locoregional failure, and then preoperatively for his third locoregional failure, suffered from chronic small bowel obstructions. The only fistula that was documented in our series occurred in a patient who had 5 metachronous locoregional failures, and who was radiated preoperatively with gross total resection of her fourth and later fifth locoregional failures.

A total of 16 patients reported grade 1-2 pain or abdominal discomfort during radiation therapy—11 in the preoperative group and 5 in the postoperative group (*P* = .47). As these tumors are often symptomatic with pain at presentation, a multifactorial cause of the pain/discomfort was likely. Narcotics were used judiciously in these patients. Those patients treated postoperatively had more frequent acute grade 1-2 genitourinary-related toxicities, when compared to the preoperative cohort (*P* = .05). There was no statistically significant difference in wound healing and/or infection in the two different cohorts—3 patients in the preoperative group and 1 patient treated postoperatively developed abscesses more than 90 days after radiation that required drainage (*P* = .31). Radiation-induced menopause was seen in 3 patients—2 treated preoperatively, and one in the postoperative setting.

## 4. Discussion

Feng et al. reported on a large group of 85 patients with RPS radiated over a 20-year period [11]. Their 2-year LC, DDFS, and OS rates were 66%, 62%, and 70%, compared to our 77%, 70%, and 70%, respectively. They were the first to report an apparent dose response relationship with adjuvant radiation therapy for RPS, with higher doses ( ≥55.8 Gy) being associated with improved LC, but with no effect on overall survival [11]. Typically, doses of 45 Gy to 50.4 Gy have been utilized in the preoperative setting for RPSs [9, 10, 12, 13]. Our 2-year rate of LRC may be, in fact, due to the higher doses of radiation used (median dose, 59.4 Gy) which may have an impact on DDFS. We routinely used this higher dose in the preoperative setting, as we have extrapolated from our own institution's data for extremity soft tissue sarcomas, with all patients—both those treated pre- and postoperatively—receiving a median dose of 59.4 Gy [27].

A direct comparison of treatment outcomes following combined surgery and radiation therapy for patients with retroperitoneal sarcomas is particularly difficult as there is no standardized definition of what is a local failure. Some reports, like our study, define a local failure as any recurrence of disease in the abdominal cavity, save the hepatic parenchyma [9]. Other reports use more stringent definitions and divide locoregional failure into “central” (within the IORT field), “local” (if within the EBRT field), “regional” (if in regional lymphatics), “peritoneal” (if seeding occurred), and “distant” (if beyond the regional site) [10]. In our series, we had no central or local failures. The predominant mode of failure was regional, but not within lymph nodes.

Our series reports a high rate of gross total resections. Twenty-six (84%) patients—fifteen treated with preoperative radiation, and 11 treated postoperatively—were able to undergo a GTR. On presentation, 2 patients were deemed by our surgeons to have technically unresectable tumors. Both were treated with preoperative radiation—one subsequently had his tumor respond enough to undergo a GTR. Both patients with technically unresectable disease eventually developed pulmonary metastases. The patient who had a GTR also had 2 separate metastectomies of lung metastases and remained without evidence of further recurrent disease 29 months after initial treatment.

We advocate a stringent follow-up regimen for these patients. The National Comprehensive Cancer Network (NCCN) guidelines recommend following patients every 3 to 6 months for the first 2 to 3 years with CTs of the abdomen and pelvis (with consideration of chest imaging) [28]. We routinely recommend CTs also of the chest every 3 and 4 months for the first 2 years, as we have seen that all patients who develop metastatic disease do so within the first 15 months of therapy. This regimen allows for identification of patients who might be candidates for initial metastectomy for oligometastases or systemic therapy at an earlier time for multiple pulmonary lesions. While there may be an element of lead-time bias with this imaging regimen, we believe that, with early identification of metastases, we can prolong disease-free intervals, while the distant disease burden is still manageable.

Many patients with RPS ultimately fail distantly and systemic therapies have not proven to be efficacious. One potential future direction in the treatment of RPSs mirrors the targeted therapy revolution currently occurring with various malignancies. Fifteen of 31 patients' tumors were stained for the presence of CD-117 (c kit) and of the 5 patients tumors that demonstrated varying degrees of positivity by immunohistochemistry, one patient received 4 months of imatinib mesylate in the preoperative setting during her radiation therapy. However, repeat immunohistochemistry for CD-117 was negative on the resected residual mass and no post-operative imatinib mesylate was given. This patient remains clinically and radiographically disease-free at 40 months.

As our results demonstrate, if a patient is to develop metastatic disease, he is at highest risk within the first years and a half after treatment. Chemotherapy might have a role in the early setting, and not just be reserved for patients who have already developed metastatic disease. Pisters et al. evaluated the use of doxorubicin concurrent with radiation therapy, and found that one could escalate to a dose of 50.4 Gy safely [13]. However, Glenn et al. from the National Cancer Institute reported increased acute toxicities with the use of postoperative triple chemotherapy (including doxorubicin) during radiotherapy in patients with resectable RPSs, without an overall survival benefit [14].

Overall, the acute toxicities associated with the modern day use of pre- or postoperative radiation therapy were mild. Thirty of 31 patients completed radiation therapy as prescribed. The patient who did not complete radiation therapy had initially presented with metastatic disease and small bowel obstruction secondary to his large tumor. He ultimately had palliative bypass surgery and succumbed to his distant disease. There were three grade 3-4 acute radiation-related toxicities (all 3 in the preoperative group), and six grade 3-4 late GI toxicities (3 in the preoperative group, 3 in the postoperative group), with the formation of only one treatment related bowel fistula. For comparison, Peterson et al. reported 12 cases of grade 3 or higher GI complications in her series of 87 (43 with primary disease, and 44 with recurrent disease) patients with RPS; 7 of these patients had fistula formation [10]. In our series, 6 patients (4 in the preoperative group, 2 in the postoperative group) also experienced late grade 3-4 infectious complications, requiring drainage of retroperitoneal abscesses and prolonged intravenous antibiotics. Grading radiation-related toxicities in patients with RPS is especially difficult, and even more so in patients who present with extremely large and/or recurrent tumors. Often times multiple abdominal surgeries have been performed—a fact alone that dramatically raises the incidence of late toxicities. There is some toxicity that must be assumed when treating these large tumors—without radiation, the risk of local recurrence is unacceptably high, and the likelihood of similar toxicities secondary to tumor growth alone is similarly high.

The use of IORT has been shown to increase local control, without adding significantly to acute and late toxicities from surgery [9, 10, 12, 16, 18, 19]. However, peripheral neuropathy has been documented with the clinical and experimental use of IORT to doses of ≥20 Gy [29, 30]. Importantly, the incidence of IORT-related peripheral neuropathy is not increased with the use of external beam radiation therapy [29, 30]. In our cohort of patients, only one patient experienced a late grade 2 neuropathy. This patient received preoperative radiation and IORT for her fourth and fifth locoregional failures. IORT doses of 10 to 12 Gy as utilized in our study almost uniformly preclude the development of late neurologic sequelae. However, IORT did not appear to significantly impact LRC, DDFS, or OS in our patients.

The American College of Surgeons Oncology Group attempted a phase III trial, which randomized patients with resectable RPS to surgery alone versus preoperative radiation therapy followed by surgical resection [31]. However, even with excellent planning and organization, the study was closed because of slow accrual. Thus, a definitive answer from a prospective randomized trial regarding the efficacy of preoperative radiation will not be available in the foreseeable future. Future studies/trials may need to include randomization of patients to surgery alone, preoperative radiation with surgery, and surgery with postoperative radiation therapy. Adding chemotherapy in the neo- or adjuvant setting, so as to reduce the risk of developing metastatic disease, can also be considered.

In summary, high dose (59.4 Gy) preoperative or postoperative radiation therapy is safe and efficacious in the treatment of RPSs. Based upon our experience, we recommend the consideration of either neoadjuvant or adjuvant radiation in patients with retroperitoneal sarcoma, as well as the possibility of IORT if there is a high likelihood of residual microscopic or minimal gross disease at the time of surgical resection. We also recommend routine use of follow-up chest CT scans along with abdominal and pelvic CT scans. Some patients who initially present with locally recurrent disease are able to have long-term disease free intervals when treated with radiation therapy at the time of initial recurrence. However, as patients who receive radiation more than one time for their RPS recurrences, the severity of adverse related events increases. Further research needs to be done, including the development of better systemic agents to combat these difficult-to-treat tumors.

## Figures and Tables

**Table 1:** Patient and tumor characteristics (all patients).

Parameter	No. of patients (%)
Sex	

Male	11 (35%)
Female	20 (65%)

Age (range)	56 (20–83)

Race	

Caucasian	28 (90%)
African American	3 (10%)

History of tobacco use	11 (35%)

Family history of malignancy	7 (23%)

Breast cancer	4 (13%)
Breast and ovarian cancers	2 (6%)
Sarcoma	1 (3%)

Prior cancer	4 (13%)

Breast	1 (3%)
Melanoma	1 (3%)
Hodgkin's disease	1 (3%)

Prior radiation	3 (10%)

TNM stage	

T2bN0M0	25 (81%)
T2bN0M1	4 (13%)
Desmoid	2 (6%)

Grade	

1	6 (19%)
2	6 (19%)
3	17 (55%)

AJCC stage	

I	10 (32%)
III	15 (48%)
IV	4 (13%)

Recurrent RPS	7 (23%)

Pretreatment size of RPS (range)	10.5 cm (4.0–20.0)

Histology	

Leiomyosarcoma	15 (48%)
Liposarcoma	10 (32%)
*Myxoid*	3 (10%)
*Dedifferentiated*	4 (13%)
Desmoid	2 (6%)
Malignant fibrous histiocytoma	3 (10%)
Inflammatory myofibroblastic sarcoma	1 (3%)

CD 117 staining	

Not done	16 (52%)
Negative	10 (32%)
Positive	5 (16%)
Imatinib mesylate given	1 (3%)

Symptoms at presentation	

Pain/discomfort	20 (65%)
Weight loss (range)	9 (31%) 10–30 lbs
Early satiety/decreased appetite	6 (21%)
Nausea	3 (10%)
Change in menses	2 (7%)
Small bowel obstruction	2 (7%)

**Table 2:** Patient, tumor, and treatment characteristics.

Parameters	Preoperative EBRT group (*n* = 19)	Postoperative EBRT group (*n* = 12)	*P* value
Gender			

Female	12 (63%)	8 (67%)	1.00
Male	7 (37%)	4 (33%)	

Age at presentation			

≤50 years	8 (42%)	2 (17%)	.24
>50 years	11 (58%)	10 (83%)	

AJCC stage			

I	5 (26%)	5 (42%)	.24
III	12 (63%)	3 (25%)	
IV	2 (11%)	2 (17%)	

Grade			

1	3 (16%)	3 (25%)	.66
2	4 (21%)	2 (17%)	
3	12 (63%)	5 (42%)	

Tumor size			

≤10 cm	7 (37%)	9 (75%)	.07
>10 cm	12 (63%)	3 (25%)	

Lesion presentation			

Primary	17 (89%)	7 (58%)	.08
Recurrent	2 (11%)	5 (42%)	

Histology			

Leiomyosarcoma	9 (47%)	6 (50%)	.72
Liposarcoma	7 (37%)	3 (25%)	
Other	3 (16%)	3 (25%)	

Extent resection			

STR	4 (21%)	1 (8%)	.62
GTR	15 (79%)	11 (92%)	

Organs removed			

No	9 (47%)	3 (25%)	.27
Yes	10 (53%)	9 (75%)	

Chemo			

No	16 (84%)	9 (75%)	.65
Yes	3 (16%)	3 (25%)	

IORT			

No	6 (32%)	9 (75%)	.03
Yes (1 pt each arm had 2 fields)	13 (68%)	3 (25%)	

Radiotherapy dose			

EBRT median (range)	59.4 Gy (36.8–63.4 Gy)	59.4 Gy (54–68.4)	.79
IORT median (range)	11 Gy (10–12 Gy)	11 Gy (10–12 Gy)	

IMRT			

No	17 (89%)	4 (33%)	.002
Yes	2 (11%)	8 (67%)	

Time to complete EBRT Median (range)	45 days (17–90 days)	44 days (41–55 days)	.76

Length of F/U Median (range)	22 months (0.6–56.5 months)	13.05 months (1.4–66.4 months)	.55

EBRT = external beam radiation therapy; STR = subtotal resection; GTR = gross total resection; IORT = intraoperative radiation therapy; IMRT = intensity modulated radiation therapy; F/U = follow-up.

**Table 3:** Cox regression multivariate analysis when controlled for pre- versus postoperative treatment.

	Locoregional control	Distant disease free survival	Overall survival
Age	*P* = .98	*P* = .79	*P* = .81
Gender	*P* = .76	*P* = .12	*P* = .72
Stage	*P* = .95	*P* = .21	*P* = .99
Grade	*P* = .81	*P* = .57	*P* = .93
Tumor size	*P* = .76	*P* = .46	*P* = .96
Primary versus recurrent	*P* = .60	*P* = .27	*P* = .65
Histology	*P* = .70	*P* = .53	*P* = .92
GTR/STR	*P* = .55	*P* = .41	*P* = .77
IORT	*P* = .55	*P* = .42	*P* = .76
Chemo	*P* = .83	*P* = .39	*P* = .77
EBRT dose	*P* = .79	*P* = .28	*P* = .60
Organ resected	*P* = .85	*P* = .34	*P* = .66

GTR = gross total resection; STR = subtotal resection; IORT = intraoperative radiation therapy; EBRT = external beam radiation therapy.

**Table 4:** RTOG acute and late toxicities of radiation.

Parameters	Preoperative EBRT group (*n* = 19)	Postoperative EBRT group (*n* = 12)	*P* value
Acute (≤90 days)			

GI			

Grade 1-2	16 (84%)	11 (92%)	.70
Grade 3-4	1 (5%)	0 (0%)	

GU			

Grade 1-2	0 (0%)	3 (25%)	.05
Grade 3-4	0 (0%)	0 (0%)	

Wound complications			

Grade 1-2	0 (0%)	1 (8%)	.39
Grade 3-4	0 (0%)	0 (0%)	

Infection			

Grade 1-2	0 (0%)	0 (0%)	1.00
Grade 3-4	1 (5%)	0 (0%)	

Skin			

Grade 1-2	2 (11%)	1 (8%)	1.00
Grade 3-4	0 (0%)	0 (0%)	

Pain			

Grade 1-2	11 (58%)	5 (42%)	.47
Grade 3-4	0 (0%)	0 (0%)	

Neurologic			

Grade 1-2	2 (11%)	0 (0%)	.51
Grade 3-4	0 (0%)	0 (0%)	

Gynecologic			

Grade 1-2	0 (0%)	0 (0%)	1.00
Grade 3-4	1 (5%)	0 (0%)	

Late (>90 days)			

GI			

Grade 1-2	1 (5%)	2 (17%)	.51
Grade 3-4	3 (16%)	3 (25%)	

GU			

Grade 1-2	0 (0%)	0 (0%)	1.00
Grade 3-4	1 (5%)	0 (0%)	

Wound complications			

Grade 1-2	0 (0%)	0 (0%)	1.00
Grade 3-4	1 (5%)	0 (0%)	

Infection			

Grade 1-2	0 (0%)	0 (0%)	.31
Grade 3-4	4 (21%)	2 (17%)	

Skin			

Grade 1-2	0 (0%)	0 (0%)	NS
Grade 3-4	0 (0%)	0 (0%)	

Pain			

Grade 1-2	1 (5%)	0 (0%)	1.00
Grade 3-4	0 (0%)	0 (0%)	

Neurologic			

Grade 1-2	1 (5%)	0 (0%)	1.00
Grade 3-4	0 (0%)	0 (0%)	

Gynecologic			

Grade 1-2	0 (0%)	0 (0%)	1.00
Grade 3-4	2 (11%)	1 (8%)	

Lymphedema			

Grade 1-2	1 (5%)	0 (0%)	1.00
Grade 3-4	0 (0%)	0 (0%)	

RTOG = Radiation therapy oncology group; EBRT = external beam radiation therapy; GI = gastrointestinal; GU = genitourinary.
